# Evidence for Enhanced Characterization of Cortical Bone Using Novel pQCT Shape Software

**DOI:** 10.1016/j.jocd.2010.05.005

**Published:** 2010-07

**Authors:** Margaret Ann Laskey, Stephanie de Bono, Daan Zhu, Colin N. Shaw, Peter J. Laskey, Kate A. Ward, Ann Prentice

**Affiliations:** 1 Nutrition and Bone Health, MRC Human Nutrition Research, Cambridge, UK; 2 Confocal Images Analysis Laboratory, MRC National Institute of Medical Research, London, UK; 3 Leverhulme Centre for Human Evolutionary Studies, University of Cambridge, Cambridge, UK; 4 Calcium. Vitamin D and Bone Health, MRC Keneba, The Gambia

**Keywords:** pQCT, tibia, bone shape, Gambia, young women

## Abstract

Bone shape, mass, structural geometry, and material properties determine bone strength. This study describes novel software that uses peripheral quantitative computed tomography (pQCT) images to quantify cortical bone shape and investigates whether the combination of shape-sensitive and manufacturer's software enhances the characterization of tibiae from contrasting populations. Existing tibial pQCT scans (4% and 50% sites) from Gambian (n = 38) and British (n = 38) women were used. Bone mass, cross-sectional area (CSA), and geometry were determined using manufacturer's software; cross-sectional shape was quantified using shape-sensitive software. At 4% site, Gambian women had lower total bone mineral content (BMC: −15.4%), CSA (−13.4%), and trabecular bone mineral density (BMD: −19%), but higher cortical subcortical BMD (6.1%). At 50% site, Gambian women had lower cortical BMC (−7.6%), cortical CSA (−12.6%), and mean cortical thickness (−15.0%), but higher cortical BMD (4.9%) and endosteal circumference (8.0%). Shape-sensitive software supported the finding that Gambian women had larger tibial endosteal circumference (9.8%), thinner mean cortical thickness (−26.5%) but smaller periosteal circumference (−5.6%). Shape-sensitive software revealed that Gambian women had tibiae with shorter maximum width (−7.6%) and thinner cortices (−22% to −41.2%) and more closely resembled a circle or ellipse. Significant differences remained after adjusting for age, height, and weight. In conclusion, shape-sensitive software enhanced the characterization of tibiae in 2 contrasting groups of women.

## Introduction

Bone shape and other aspects of structural geometry, mass, and material properties together determine bone strength. Bone geometry describes the distribution of skeletal mass from the centroid. The farther the centroid is from the skeletal mass, the greater is the bone's ability to resist bending deformation *(1)*. The genetic makeup and lifestyle of a subject, especially skeletal loading, contribute to the strength of an individual bone. Quantifiable measures of bone mass and size can be obtained using dual-energy X-ray absorptiometry (DXA) and peripheral quantitative computed tomography (pQCT). DXA has been considered the “gold standard” for measurements of bone mineral status, and the World Health Organisation's criteria for defining osteoporosis are based on DXA T-score values of areal bone mineral density (aBMD) *(2)*. However, this approach to osteoporosis diagnosis fails to identify many individuals who eventually sustain fragility fractures *(3,4)*. DXA measurements are now recognized to have several limitations *(5)*. pQCT has the advantage that it measures the true volumetric density (BMD) of trabecular and cortical bone separately. In addition, pQCT creates cross-sectional images of bone, from which the cross-sectional area (CSA) of bone, and hence structural geometry, can be determined. Using these pQCT images, it was possible to observe distinct qualitative differences in cross-sectional bone shape from 2 different populations with contrasting lifestyles. Unfortunately, currently available manufacturer's software does not quantify in detail the cross-sectional shape of these images.

The aim of this study was to devise novel software to enable the irregular cross-sectional shape of cortical bone to be quantified using cross-sectional bone images generated from pQCT scans. The new software was then used, in combination with the manufacturer's software, to compare preexisting data sets of pQCT scans from young Caucasian women living in the affluent British University town of Cambridge, UK, and scans from young Mandinka women living in poor, rural villages in The Gambia. These populations have very different genetic backgrounds, habitual biomechanical loading patterns, and nutritional status. The skeletal site chosen for this comparison was the tibia, as this is one of the predominantly loaded sites. The aim was to investigate whether the new shape-sensitive software would enhance the discrimination between tibiae from these contrasting populations. Based on previous qualitative observations, it was anticipated that the 2 groups of women would have different bone geometry and bone shape.

## Materials and Methods

### Peripheral Quantitative Computed Tomography

pQCT measurements of the left tibia were carried out using Stratec XCT-2000 scanners (Stratec, Medizintechnik GmbH, Pforzheim, Germany) located in Cambridge, UK, and Keneba, The Gambia. All scans were analyzed in Cambridge using Stratec software version 6.00B. Tibial length was measured to the nearest 0.5 cm using a tape measure. Tibial length was defined as the distance from the midpoint of the medial malleolus to the medial aspect of the tibial plateau.

A scout view of the distal end of the tibia allowed the positioning of the reference line at the middle of the distal end plate. From this reference line, the machine located the 2-mm-thick measurement sites at 4% and 50% of the tibial length. The voxel size for all measurements was 0.5 mm. The 4% site contains predominantly trabecular bone and the 50% site mainly cortical bone. At the 4% site, trabecular bone was defined as the inner area of 45% of the total CSA, to exclude the remaining 55% of bone containing some trabecular bone and the cortical shell. The latter was defined as cortical sub-cortical bone. At the 4% tibial site, total bone mineral content (Tot BMC, mg/mm), total CSA (Tot CSA, mm^2^), total volumetric bone mineral density (Tot BMD, mg/cm^3^), trabecular BMD (mg/cm^3^), and cortical subcortical BMD (mg/cm^3^) were determined. For these distal-site measurements, the bone threshold was set at 180 mg/cm^3^ (contour mode 1 and peel mode 1).

At the 50% tibial site, the measurements of interest were Tot CSA (mm^2^), cortical BMC (mg/mm), cortical CSA (mm^2^), cortical BMD (mg/cm^3^), cortical thickness (mm), periosteal circumference (mm), endosteal circumference (mm), strength strain index (SSI, mm^3^), muscle CSA (cm^2^), fat CSA (cm^2^), and Tot bone CSA (tibia plus fibula, cm^2^). Cortical BMD and all bone geometry measurements were determined using the threshold of 710 mg/cm^3^ and separation mode 1. Manufacturer's software provides 2 estimates of cortical thickness, periosteal and endosteal circumferences. One software assumes that bones are circular (circular ring model) and the other does not (noncircular model). Only data from the circular model are presented, as the manufacturers only recommend the use of this model. The SSI (mm^3^) was determined using the bone threshold of 480 mg/cm^3^. SSI is the density-weighted polar section modulus of a cross-section and reflects the strength of the long bone with respect to torsion (multiplane bending). Muscle CSA was determined using contour mode 3, peel mode 1, filter F03F05, and the threshold of 40 mg/cm^3^. Fat tissue CSA was determined using contour mode 3, peel mode 1, and a threshold of −53 mg/cm^3^.

The respective reported measurements (coefficient of variation %) for pQCT measurements at the tibia in Cambridge (n = 30) and Keneba (n = 31) (2 repeat measures) are as follows: 4% site—trabecular BMD = 0.9%, 1.2%; Tot BMD = 1.1%, 0.9%; 50% site—cortical BMD = 0.7%, 0.4%; cortical CSA = 1.5%, 0.8%; 50% site muscle CSA = 1.6%, 3.3%. The quality assurance for pQCT was performed on all working days in both Cambridge and Keneba. The trabecular attenuation and Tot CSA determined for the standard and cone phantoms were always within the accepted tolerance for both pQCT systems. All pQCT scans were scrutinized for movement artifacts and other potential problems to ensure that the scans were of sufficient quality to be included in the study.

### Cortical Shape-Sensitive Software

The software was designed to quantify the differences in bone shape that had been observed in pQCT images at the 50% tibial site from Gambian and British women. This software will be available without cost for academics for research purposes (www.mrc-hnr.cam.ac.uk).

#### Determining the Accuracy of Exported Images

To ensure that the pQCT output files (.raw) were accurate representations of the bones being scanned, raw pQCT images from dry human skeletal remains were compared with CSA images obtained from latex molds of the same bones. Images (voxel size: 0.5; image type: 16 bit; height × width: 283 × 283 pixels; 2.02 pixels = 1 mm) were obtained from pQCT scans of 9 dry humeri and 10 tibiae from human skeletal remains selected, with permission, from the Duckworth Collection, Leverhulme Centre for Human Evolutionary Studies, University of Cambridge. Latex cast moldings were taken from the same bones. The molds were constructed using the “latex cast method,” a highly accurate technique for replicating CSA of long bones *(6)*. Cross-sectional images of the latex casts were obtained using a flatbed scanner. The bone images derived from the latex casts and pQCT images were imported into Image J (US National Institutes of Health, http://rsb.info.nih.gov/ij/). Tot CSA for all images (pQCT derived and latex mold derived) were calculated in Image J using Moment Macro v 1.4 (http://www.hopkinsmedicine.org/FAE/mmacro.htm). There were no significant differences (*p* = 0.9) in Tot CSA for the images derived from the latex casts and those derived using the raw pQCT images analyzed with Moment Macro (mean Tot CSA: latex cast = 348 mm^2^, standard deviation [SD] = 108; raw pQCT image = 350 mm^2^, SD = 107; *r* = 0.99, constant = 6.6, standard error (SE) = 11.0; coefficient = 0.99, SE = 0.03; *p* < 0.001). In addition, there was no significant difference (*p* = 0.7) in Tot CSA of these bones when images were derived from latex casts or when the bones were analyzed using the manufacturer's software (mean Tot CSA: latex mold = 348 mm^2^, SD = 108; raw pQCT image/manufacturer's software = 363 mm^2^, SD = 110, *r* = 0.99, constant = 9.0, SE = 9.7; coefficient = 1.0, SE = 0.03, *p* < 0.001). It was not possible to use JPEG or BMP images generated from the software, as these images had been modified for display purposes only.

#### Shape Software

The new software was a customized plugin for Image J. This program took into account the irregular cross-sectional shape of the tibia. The software allowed for the threshold to be modified (200–1000), enabling the optimum threshold to be selected for a specific investigation and data set. The voxel size was then selected to correspond with the scan voxel size. The region of interest within the bone image (cortical bone) was selected manually using the Image J crosshair tool. A “flooding algorithm” then automatically identified neighboring pixels of similar intensity to define cortical bone. After the cortical bone had been identified, the software used erosion and dilation to remove background noise and identify the endosteal and periosteal boundaries.

The analysis estimated cortical bone distribution throughout the cross-section, relative to the center of mass. This was accomplished using an “edge coding algorithm,” which output variables related to bone shape (maximum diameter, minimum diameter [defined as at right angles to maximum diameter] and periosteal and endosteal circumferences). The software also calculated the angle of maximum diameter from the vertical axis (angle data not reported in this article). A visual representation of all outputs was obtained, and these were used to ensure that a bone has been correctly analyzed. When there were obvious movement artifacts in the scan image, the program usually failed, and it was obvious that the data were invalid and should be excluded.

For the data sets described in this article, the voxel size of 0.5 and threshold of 450 were selected. This threshold was chosen as the visual representations from the outputs revealed that the threshold of 450 included the fairly dense trabeculization on the endosteal surface. This trabeculization was clearly visible in about a third of the scans. Cortical thickness was calculated by measuring the distance from the periosteal boundary to the endosteal boundary. Anterior and posterior cortical thickness was calculated along the axis of maximum diameter. For other measures of cortical thickness (maximum, minimum, mean, left, right), the thickness was determined as the shortest distance between the periosteal and the endosteal boundaries. For clarity, only anterior, posterior, minimum, and mean cortical thicknesses were reported for this study.

The shape-sensitive software also quantified how far the CSA of a bone deviated from an ideal circle and/or ellipse (average error from an ideal circle and ellipse). The fitting of an ideal circle and ellipse was based on the calculations of Fitzgibbon et al. *(7)*. The smaller the error value the closer the cross-section of a bone was to the ideally calculated circle or ellipse. Hence, a bone that was perfectly circular (elliptical) in cross-section had an error value of 0.

### Data-Sets

Good-quality tibial pQCT scans from preexisting data sets were used to investigate the potential of the novel shape-sensitive software. The pQCT scans were of young women living in Cambridge, UK, and in a rural region of The Gambia. The scans selected for the current investigation were from women aged between 24 and 36 yr. This age range was chosen to maximize the likelihood that all women had stopped growing and were not perimenopausal.

The Caucasian women living in Cambridge, UK, were a subset of healthy, nonpregnant, nonlactating women participating in a larger, longitudinal study designed to investigate the impact of pregnancy and lactation on bone. The Cambridge Local Research Ethics Committee approved the study, and subjects gave written informed consent. Full details of this study, inclusion and exclusion criteria have been described previously *(8)*. The subset of 38 Cambridge women included in this study were selected if they had tibial pQCT, height and weight measurements, had not been pregnant within the previous 2 yr, and had not lactated within the previous year. Thirty of these women were nulliparous and 8 were of parity 1–3.

The Gambian women were born in the rural West Kiang villages of Keneba, Kantong Kunda, or Manduar, The Gambia, and were participating in a larger study designed to investigate early-life predictors of low-grade systemic inflammation. Women were selected for the main study if they were healthy and not suffering from a serious or chronic illness, were not pregnant, and were registered in the Keneba Antenatal Scheme; hence, complete birth details were available. The study was approved by The Gambian Government/MRC Laboratories Joint Ethics Committee and by the Ethics Committee at the London School of Hygiene and Tropical Medicine. Informed consent was obtained from all women. The subset of 38 Gambian women included in this study was selected if they had tibia pQCT, height, and weight measurements completed. Four of the women were nulliparous, 19 were of parity 1–3, and 15 were of parity 4–5. Twenty of the women were lactating (2–20 mo postpartum) at the time of their pQCT measurement.

### Statistical Analyses

Data Desk 6.1.1 (Data Description Inc., Ithaca, NY) software was used for all analyses. Descriptive statistics are reported as median and interquartile ranges to demonstrate the distributions of the skeletal measurements. Linear regression analysis was used to compare results for mean cortical thickness and for endosteal and periosteal circumferences determined using the shape-sensitive and manufacturer's software *(9)*. To investigate group differences, all continuous variables, except age, were transformed to natural logarithms before analysis to normalize skewness and to investigate proportional effects *(10)*. When the dependent variable is in natural logarithms, the multiplication of the regression coefficient by 100 determines the sympercent difference *(11)*. Skeletal differences between the groups (Gambian/Cambridge: 1/0) were determined using linear regression with and without correction for age, height, and weight. The percent differences between Gambian and Cambridge women, at both the 4% and 50% sites, were determined. In addition, at the 50% tibial site only, multiple regression models were set up with the following independent variables: age, height, weight, tibial length, muscle CSA, and fat CSA. Results are reported for the full models. Significant independent variables are identified. To consider the potential impact of lactation on results, analyses were also performed with (n = 38) and without (n = 18) the 20 lactating Gambian women.

## Results

### Subject Characteristics

The characteristics of the Cambridge and Gambian women are shown in Table 1. The Gambian women were significantly shorter, lighter, and younger than the Cambridge women. However, the Gambian women had significantly longer tibia. There was no significant difference between the 2 groups at the 50% site in Tot bone CSA (tibia and fibula), but CSA of fat, muscle, and total leg (sum of bone, fat, muscle) were more than 30% smaller at the 50% tibial site for the Gambian women (Table 1). There were no significant differences in % fat (Gambian women: median = 36.2%, interquartile range = 33.7–40.0; Cambridge women: median = 37.9%, interquartile range = 30.9–41.8; *p* = 0.8).

### Trabecular Site (4%)

The results for the tibial 4% trabecular site are presented in Table 2. Tot BMC, Tot CSA, and trabecular BMD were significantly lower for the Gambian women, but cortical and subcortical BMD was significantly greater. However, after adjusting for height, weight, and age, the differences in Tot BMC and Tot CSA were not significant. However, the difference in trabecular BMD (−20.5%) remained significant. Age was not a significant predictor of the group differences for any tibial measurement. When only data from nonlactating Gambian women (n = 18) were included in the analyses, the group differences were similar to those observed when all the Gambian women were included (n = 38). For example, when only nonlactating women (n = 18) were included, the Gambian women had 16.4% (*p* < 0.001) lower trabecular BMD but 8.4% (*p* < 0.05) greater cortical and subcortical BMD. The significance of the differences tended to be smaller when lactating women were excluded from the analysis, probably reflecting the smaller sample size.

### Cortical Site (50%)

pQCT cortical bone outcome variables for the 50% tibial site are shown in Table 3. The Gambian women had significantly lower cortical BMC, CSA, mean cortical thickness, but greater cortical BMD and endosteal circumference than the Cambridge women. After adjustments for height, weight, and age, differences between groups for cortical BMC and cortical CSA were not significant. However, Tot CSA, periosteal circumference, and SSI became significantly greater for the Gambian women after adjustment.

Additional analyses were performed after adjusting for tibial length and CSA of fat and muscle at the 50% site, as well as age, weight and height (results not presented). Fat CSA and tibial length, but not muscle CSA, were significant independent predictors in some but not all analyses. In general, the percent differences between the groups using this model were similar to the adjusted results presented in Table 2. Results were also similar when only the nonlactating Gambian women (n = 18) were included in the analysis (data not shown).

### Shape-Sensitive Software Results for 50% Cortical Site

Table 3 presents the results obtained using the novel shape-sensitive software. Both manufacturer's pQCT software and the shape-sensitive software provide measures of periosteal and endosteal circumference and also mean cortical thickness. The shape-sensitive software supports the findings presented in Table 2, which demonstrated that the Gambian women had significantly larger endosteal circumferences (and hence larger medullary cavity) but smaller mean cortical thickness. However, the shape-sensitive software values for the 2 groups of women were significantly greater for periosteal circumference (23–27%, *P* < 0.001) and endosteal circumference (16–18%, *p* < 0.001) than those determined using the manufacturer's software. The correlations between the results obtained using the manufacturer and shape-sensitive software methods were high. Agreement was best for the periosteal circumference of the Gambian women (*r* = 0.96; constant = 3.7, SE = 3.1; coefficient = 0.75, SE = 0.03; *p* < 0.0001) and less good for endosteal circumference of the Cambridge women (*r* = 0.78; constant = 18, SE = 3.0; coefficient = 0.46, SE = 0.06; *p* < 0.0001).

For the Gambian women, there was no significant difference in mean cortical thickness determined by the different software, and there was a high correlation between the 2 sets of results (*r* = 0.97; constant = 0.76, SE = 0.15; coefficient = 0.85, SE = 0.03; *p* < 0.0001). In contrast, for the Cambridge women, the 8% greater cortical thickness determined by the shape-sensitive software was significant (*p* < 0.001), and the agreement between the 2 methods was less good (*r* = 0.8; constant = 1.9, SE = 0.4; coefficient = 0.57, SE = 0.07; *p* < 0.0001).

The shape-sensitive software also revealed that the tibiae of the Gambian women had significantly smaller maximum diameter and cortical thickness (anterior, posterior, and minimum) but similar minimum diameter. In addition, compared with the tibiae of the Cambridge women, the shape of Gambian tibiae more closely resembles a circle and ellipse. After size adjustment, many of these differences remained. Results were also similar after further adjustment for tibial length and fat and muscle CSA (data not shown).

**Fig. 1:**
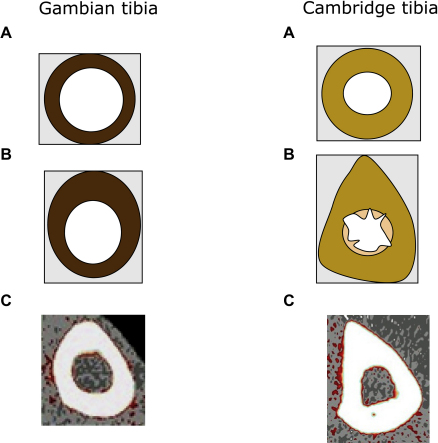
Cartoon representations of tibiae at 50% site from the Gambian and Cambridge women using (**A**) data derived from manufacturer's software (Table 3) and (**B**) using data derived from manufacturer's software and shape-sensitive data (Table 4) combined. For comparison, actual peripheral quantitative computed tomography (pQCT) images, typical of each of the groups, are shown (**C**). The cartoons are not drawn to scale. Darker colors are used to represent more dense bone.

The differences between the tibiae from Gambian and Cambridge women, at the 50% site, are illustrated in Fig. 1. These cartoon figures were prepared using information obtained from manufacturer's software (Table 2) and the novel shape-sensitive software (Table 3). The figure demonstrates the additional information that the new shape-sensitive software provides. This includes the data about variations in cortical thickness, maximum and minimum diameters, and average error from ellipse. For comparison, typical pQCT tibial images of the 50% site from the Gambian and Cambridge women are included in Fig. 1.

## Discussion

This article describes the development and procedure for use of novel software that was designed to quantify the cross-sectional shape of tibial cortical bone using pQCT images. One important feature of the new software is the automatic visual representation of all outputs. This allows immediate identification of any program malfunction, ensures invalid data are rejected immediately, and also facilitates selection of the optimum threshold for a specific study.

The comparison of tibiae from the Gambian and Cambridge women demonstrated that group differences in BMD and CSA could be identified using manufacturer's software (Tables 1 and 2), but that use of shape-sensitive software enabled additional differences, related to bone shape, to be identified (Table 3). None of the differences could be explained by correcting for age or body composition and, only in a few cases, by the lower weight and shorter stature of the Gambian women (Tables 1–3).

Both the shape-sensitive and manufacturer's software revealed that, after size correction, the Gambian women had, on average, a 16% larger endosteal circumference than the Cambridge women, but differences in periosteal circumference were small. However, different assumptions are used for the measurement of bone circumference by the different software. The shape-sensitive software takes into account the irregular contours of the tibiae. In contrast, the measurements of periosteal and endosteal circumference, recommended by the manufacturer, assume that bone is circular in cross-section. Thus, it was expected that the shape-sensitive measurements would be larger than those determined using the manufacturer's software, as proved to be the case (Tables 2 and 3). It can be speculated that differences between software circumference measurements will be greatest for bones that are most irregular in cross-section.

The shape-sensitive software threshold of 450, used to determine cortical thickness, was chosen, because visual representations from the outputs revealed that this threshold included the fairly dense trabeculization on the endosteal surface that was clearly visible in about a third of the scans. This choice of threshold, therefore, allowed the investigation of degrees of trabeculization. In the Gambian data set, the results for mean cortical thickness, obtained using both softwares, were similar. In contrast, for the Cambridge data set, the shape-sensitive measurements were greater in value than those obtained with the manufacturer's software. These results suggest that the Cambridge women had greater trabeculization at the endosteal surface compared with the Gambian women. This is illustrated in Fig. 1.

The differences observed in the pQCT data-sets from Cambridge and Gambian young women used to investigate the potential of the new software are interesting, because they may be a reflection of the differences in genetics, dietary intake, vitamin D status, physical activity, and other aspects of lifestyle that have been documented in these 2 populations *(12–17)*. For example, the observed differences in bone shape may relate to contrasting skeletal loading patterns of Cambridge and Gambian women because of differences in energy expenditure: Gambian women have moderate to heavy workloads, such as regularly carrying loads on their heads (e.g., water, harvested crops) and infants on their backs *(16)*.

At the 4% tibial site, the Gambian women had significantly lower trabecular BMD but higher cortical subcortical BMD than the Cambridge women. Because the cortical subcortical region comprises both trabecular bone and the cortical shell, this suggests that the Gambian women had more cortical bone (thicker cortical shell), greater cortical BMD, or a combination of these. The finding of a 5% greater cortical BMD at the 50% site in the Gambian women supports the possibility that the difference is because of a greater cortical BMD. In addition, the greater cortical BMD of the Gambian women reinforces the evidence that measured cortical BMD is not uniform, and that it varies both within and between individuals. The degree to which differences in cortical BMD reflect variations in cortical porosity, mineralization, or a combination of these is unclear.

The SSI at the 50% tibial site is routinely used as a measure of bending strength, combining data related to the geometry of the cortical shell and cortical BMD. SSI was found to be similar in the 2 groups of women before size adjustment but was 17% greater in the Gambian women after size adjustment (Table 2). The ability of a bone to resist bending and tension is greater when the skeletal mass is located further away from the neutral axis and is proportional to the fourth power of the distance from the neutral axis *(1)*. Thus, despite the lower cortical BMC and cortical thickness of the Gambian women, their bone geometry and greater cortical BMD resulted in a greater SSI, after size adjustment, and hence, bending strength at the 50% site in the Gambian women. The more circular and symmetrical distribution of bone observed for Gambian women suggests that Gambian tibiae are adapted to the types of loading to which they are routinely subjected. This organization of bones may make them more capable of coping with unusual external loading *(18)*. Previous studies have demonstrated that low aBMD is common in Gambian women, but fragility fractures are rare *(19)*. However, the bone mineral measurements were conducted using DXA, a technique now recognized to have limitations *(5)*. The current study, therefore, has suggested that there may be differences in skeletal shape and internal structure that may contribute to the reported differences in fracture risk. Future studies may clarify this issue. This is because the displacement of skeletal mass (BMC) from the neutral axis increases the projected bone area (BA). Hence, DXA measurements of aBMD (BMC/BA) are lower but may be counterbalanced in terms of bone strength by the skeletal geometry (Fig. 1).

There are some limitations to this study. First, 20 of the 38 Gambian women were lactating at the time of their pQCT measurement. Lactation is known to decrease bone mineral at predominantly trabecular skeletal sites *(20)*. Exclusion of all the lactating Gambian women from the analyses had minimal impact on the magnitude of the group differences, even at the trabecular-rich 4% tibial site. The significance of the differences was lower probably because of the smaller sample size. Hence, it is reasonable to conclude that the observed differences in tibial bone relate to the ethnic, dietary, and lifestyle differences, and not to skeletal changes related to lactation. Second, we do not have in vivo cross-calibration data for our 2 scanners. Standard quality assurance measurements are best for checking consistency in Tot CSA and trabecular BMD but are less good for cortical BMD. We cannot, therefore, rule out technical differences to explain the differences seen in cortical BMD.

In conclusion, this study demonstrated that the data generated by the novel shape-sensitive software are robust and provide additional information on bone structural geometry that enhances the characterization of bone and, hence, improves discrimination in peripheral pQCT scans between individuals from contrasting populations. This study also provided an insight into potential ethnic differences in skeletal shape and internal structure, which may reflect skeletal adaptation to differences in genetics or lifestyle.

## Figures and Tables

**Table 1:** Subject Characteristics of the Gambian and Cambridge Young Women

	Gambian (n = 38)		Cambridge (n = 38)		% Diff^a^
Median	Interquartile range	Median	Interquartile range
Age (yr)	26.4	25.2–27.3	31.9	29.1–33.5	−17.4∗∗∗
Height (cm)	160.3	156.2–165.2	167.5	164.0–171.0	−4.5∗∗∗
Weight (kg)	54.6	49.0–59.7	63.8	57.3–70.3	−16.9∗∗∗
Tibial length (cm)	390.0	375.0–405.0	367.5	355.0–380.0	5.3∗∗∗

Body composition at 50% tibia as CSA					
Tot bone CSA (cm^2^)	518	470–562	505	483–562	−0.1
Fat CSA (cm^2^)	2092	1904–2508	3038	2380–3950	−32.7∗∗∗
Muscle CSA (cm^2^)	3141	2750–3783	4804	4366–5172	−39.9∗∗∗
Tot leg CSA (cm^2^)	5884	5218–6763	8591	7345–9574	−35.0∗∗∗

Significant differences between groups: ∗∗∗*p* < 0.001. *Abbr*: Tot bone CSA, total cross-sectional area of tibia + fibula; Tot leg CSA, total cross-sectional area of fat + muscle + total bone.

a % Diff: percentage difference between groups calculated using linear regression.

**Table 2:** Comparison of Trabecular Bone Measurements of the Gambian and Cambridge Young Women at 4% Tibia

	Gambian (n = 38)		Cambridge (n = 38)		% Diff^a^	% Diff adj^b^	Significant predictors
Median	Interquartile range	Median	Interquartile range
Tot BMC (mg/mm)	252.5	227.5–281.0	293.1	266.8–329.2	−15.4∗∗∗	−5.8	Wt∗∗∗
Tot CSA (mm2)	898.1	854.0–981.3	1032.6	963.3–1106.5	−13.4∗∗∗	−3.4	Ht∗∗∗
Tot BMD (mg/cm^3^)	281.3	258.5–309.5	292.5	268.9–306.2	−2.0	−2.4	−Ht∗, wt∗∗
Trab BMD (mg/cm^3^)	184.6	174.2–200.3	230.9	198.9–255.0	−19.0∗∗∗	−20.5∗∗∗	Wt∗∗
Cortsub Cort BMD (mg/cm^3^)	358.7	327.4–393.2	339.7	320.5–361.1	6.1∗	6.3	−Ht∗, wt∗∗

Significant predictors of group differences are shown (− indicates a negative predictor). Significance of results: ∗*p* < 0.05; ∗∗*p* < 0.01; ∗∗∗*p* < 0.001. *Abbr*: Tot BMC, total bone mineral content; Tot CSA, total tibia cross-sectional area; Tot BMD, total bone mineral density; Trab BMD, trabecular bone mineral density; Cortsub Cort BMD, cortical + subcortical bone mineral density; Wt, weight; Ht, height.

a % Diff: percentage difference between groups calculated using linear regression. Positive differences indicate that Gambian values are greater.

b % Diff adj: percentage difference between groups after adjusting for height, weight, and age.

**Table 3:** Comparison of pQCT Cortical Bone Measurements of the Gambian and Cambridge Young Women at 50% Tibia Site

	Gambian (n = 38)		Cambridge (n = 38)		% Diff^a^	% Diff adj^b^	Significant predictors
Median	Interquartile range	Median	Interquartile range
Tot CSA (mm^2^)	409.3	381.0–444.0	407.0	383.5–457.0	−1.8	8.7∗	Wt∗∗, ht∗∗
Cort BMC (mg/mm)	298.6	266.5–326.2	316.4	297.5–352.4	−7.6∗∗	0.9	Wt∗∗, ht∗
Cort CSA (mm^2^)	247.1	224.3–272.8	274.3	261.8–304.0	−12.6∗∗∗	−4.1	Wt∗∗
Cort BMD (mg/cm^3^)	1205	1189–1218	1146	1135–1159	4.9∗∗∗	5.0∗∗∗	
PeriC (mm)	71.7	69.2–74.7	71.5	69.4–75.8	−0.9	4.4∗	Ht∗∗, wt∗∗
EndoC (mm)	45.2	40.1–48.2	41.2	38.7–43.5	8.0∗∗	14.7∗∗∗	Ht∗
Mean Cort thk (mm)	4.32	3.84–4.55	4.92	4.73–5.28	−15.0∗∗∗	−12.4∗∗	Wt∗
SSI (mm^3^)	1660	1477–1857	1543	1438–1934	2.1	16.7∗∗	Ht∗∗, wt∗∗

Significant predictors of group differences are shown (− indicates inverse relationship). Significance of results indicated: ∗*p* < 0.05; ∗∗*p* < 0.01; ∗∗∗*p* < 0.001. *Abbr*: Tot CSA, total cross-sectional area; Cort BMC, cortical bone mineral content; Cort CSA, cortical cross-sectional area; Cort BMD, cortical bone mineral density; PeriC, periosteal circumference; EndoC, endosteal circumference; Cort thk, cortical thickness; SSI, strength strain index; wt, weight; ht, height.

a % Diff: percentage difference between groups calculated using linear regression. Positive differences indicate that Gambian values are greater.

b % Diff adj: percentage difference between groups calculated after adjusting for weight, height, and age.

**Table 4:** Shape Analysis Results for Gambian and Cambridge Young Women at 50% Tibia

	Gambian (n = 38)		Cambridge (n = 38)		% Diff^a^	% Diff adj^b^	Significant variable
Median	Interquartile range	Median	Interquartile range
Max diam (mm)	25.7	24.8–27.6	27.9	26.9–28.9	−7.6∗∗∗	−4.2	Ht∗, wt∗
Min diam (mm)	17.8	16.8–18.9	17.2	16.0–18.3	2.6	11.0∗∗	Wt∗
PeriC (mm)	91.5	86.5–95.0	95.0	91.5–99.5	−5.6 ∗∗∗	1.3	Ht∗∗, wt∗∗
EndoC (mm)	54.5	47.5–60.5	49.5	45.5–53.5	9.9∗∗	17.8∗∗∗	Ht∗∗
Ant Cort thk (mm)	7.62	6.80–8.25	11.42	10.69–12.30	−41.2∗∗∗	−41.1∗∗∗	
Post Cort thk (mm)	4.27	2.69–6.33	5.30	4.61–6.10	−22.2∗∗∗	−28.0∗∗∗	Wt∗∗
Min Cort thk (mm)	2.24	2.06–2.50	2.76	2.50–3.16	−21.5∗∗∗	−18.3∗∗	
Mean Cort thk (mm)	4.15	3.65–4.40	5.36	5.08–5.67	−26.5∗∗∗	−25.1∗∗∗	Wt∗∗
Av E Ellip (mm^2^)	10.5	9.1–12.5	12.03	11.6–13.0	−14.2∗∗∗	−13.3∗∗∗	
Av E Circ (mm^2^)	0.045	0.04–05	0.057	0.05–0.06	−23.1∗∗∗	−18.0∗∗	

Significance between groups and significance of explanatory variables (in brackets in column 7): ∗*p* < 0.05; ∗∗*p* < 0.01; ∗∗∗*p* < 0.001. Significant predictors after correcting for height, weight, and age are shown. Positive difference indicates that Gambian values are greater. *Abbr*: Max diam, maximum diameter; Min diam, minimum diameter; PeriC, periosteal circumference; EndoC, endosteal circumference; Ant Cort thk, anterior cortical thickness; Post Cort thk, posterior cortical thickness; Min Cort thk, minimum cortical thickness; Av E Circ, deviation of bone shape from circle; Av E Ellip, deviation of bone shape from ellipse.

a % Diff: percentage difference (sympercent) between Gambian and Cambridge women calculated using simple regression with all variable in natural logs (see Materials and Methods).

b % Diff adj: percentage difference between groups calculated using multivariate analyses after adjusting for current height, weight, and age.
